# Clinical Review, and Hospital Reports

**Published:** 1844-10-01

**Authors:** 


### Clinical Review.

### ST. GEORGE'S HOSPITAL.

Report of some Cases occurring under the Care of Mr. Henry James Johnson, Assistant Surgeon.

Some years ago, I related, in this Journal, several cases of an affection of the Deltoïd Muscle, and made some observations on its nature. Although those observations were founded on the cases I had actually witnessed, they made no claim to absolute originality, a paper being published on the same subject about that time, or just prior to it, in the Midland Journal. As the complaint does not appear to be generally understood, no apology, perhaps, will be considered due, for attention being again directed to it.

It essentially consists in loss of power, more or less complete, in the deltoïd muscle, accompanied with pain in the attempt to use it.

The arm hangs, or is supported by the side. It can be moved, or rather swung, backwards and forwards in a limited degree, but more forwards than backwards. The elbow can be carried to a small distance only from the side, the attempt to elevate it to a horizontal plane with the shoulder, being not only painful, but impracticable. The hand, of course, cannot be raised to the head, nor can it be carried back to the spine, and the want of power to effect these movements is evidently due, in some degree, to pain, felt in the deltoïd, generally at its insertion, and often felt, too, on the front and side of the humerus.

It is clear, on inspection, and indeed from this description, that the deltoïd is the muscle principally concerned. It is usually found to be attenuated, the wasting being in the ratio of the severity of the complaint or its duration. Sometimes one portion of the deltoïd is more affected than another; the central and posterior divisions suffer most. Pressure on the muscle gives some uneasiness over its insertion—it may even give pain.

But it is not, in all cases, the deltoïd alone that is at fault. Other muscles around the joint may be involved. It would be difficult to particularise them with accuracy. I have found the coraco-brachialis very obviously affected, and have thought that the biceps was implicated.

With the progress of the disease, the wasting of the deltoïd extends to the whole limb, the paralysis grows more complete, the shoulder is almost fixed, and the movements of the extremity are limited to those of the hand and fore-arm. The pain ultimately diminishes or disappears, and the use of the shoulder-joint is lost.

So far as I have seen, the subjects of this affection have commonly attained middle life, or passed it. I do not remember having seen a patient under twenty years of age. They are for the most part of a rheumatic or gouty habit, and the general health has been such as might be expected from this circumstance—not absolutely bad, nor very good.

The apparent cause of the disease, is, in most instances, exposure to wet and cold; to that, at least, the patients have attributed it. And such is probably the case, although a predisposition must, I apprehend, be looked for in a gouty or rheumatic constitution. I am led to this opinion, partly from the history of the cases themselves, and partly from this consideration:—Persons of a gouty habit frequently suffer from pain in the shoulder, aggravated by the attempt to bring the deltoïd into play, and sometimes even temporarily rendering it useless. Such persons cannot raise the arm, or carry it backwards without actual suffering.

The attack lasts for a day or two, or for a much longer period—occasionally, even, for weeks. These persons are also annoyed by fugitive pains in other parts of the body, particularly in the site of the extensor tendons, on the wrist, and on the instep. Their arthritic character is proved by the supervention, in some instances, of a fit of gout or rheumatism, as well as by the general habit of body. The transition from a slight case of this character to the paralysis of the deltoïd that I have described is by easy and obvious gradations.

It does not appear obvious why the deltoïd muscle should be peculiarly the seat of this affection. It is not more exposed to atmospheric influences than other muscles of the body, nay it is not so much exposed as some, for example, the great pectoral. As I never had the opportunity of examining the state of the limb, after death, I am unable to say what element of the muscle is *the* one affected. Is it the fascial envelope—or the interstitial and investing cellular tissue of the fasciculi—or is it the muscular fibre itself? Analogy and probability would point to the fascia and cellular membrane. One circumstance should not be lost sight of. The posterior circumflex nerve, a large one, is situated between the muscle and the bone, where it suddenly and profusely subdivides and is lost in the muscle itself. The nerve in its course, and at its subdivision, is immersed in a quantity of cellular tissue. It is difficult to suppose that this anatomical fact exercises no influence in the affection. It is *possible* that it may in some degree determine the liability of the muscle to the complaint—it is *probable* that it is the great cause of the extreme pain and the consequent paralysis. If any are disposed to doubt this explanation, they would do well to think of sciatica.

The treatment is, in the main, what would be naturally suggested by the character of the disorder, Persistent pain in any organ, aggravated by the exercise of its ordinary function, is in most cases, a sufficient reason for the employment of counter-irritation. And counter-irritation, actively and resolutely followed up, is what I have found of the greatest service. It is to blister after blister—the tartar emetic, in ointment or in plaister—and, last not least, the moxa, that we must seek, and shall find, the most efficient remedies. Colchicum and the iodide of potassium are of service, in fair and steady doses. We may begin by purging and lowering somewhat, but we must not (for it is useless) pull the patient down too much, and after a while I have found my account in the exhibition of sarsaparilla. The vapour bath, almost always of advantage in this sort of case, may be beneficially resorted to, and, when the pain has subsided, frictions with liniments containing belladonna, electricity, and passive motion should be had recourse to.

I have hitherto said nothing of the exhibition of mercury. Yet, in an early stage of the complaint, when the suffering is acute, and the system strong, calomel and opium (the latter in good quantity) may be given so as to touch the mouth.

The affection is obstinate—the treatment seems, for a time, unsatisfactory—but if carried out judiciously and patiently, it is ultimately, I believe, in most instances, successful. I was formerly less sanguine than I now am, of the issue. The real difficulty is to persuade the patient to submit, long enough, to the counter-irritation, that is requisite. That demands nerve, which some have not, and in them I should apprehend a less fortunate result than is desirable—permanent loss of power of the arm.

I subjoin one or two cases—not enough to bear out all the foregoing remarks, or furnish a clinical history of the affection, but merely those which have lately occurred, under the eyes of the pupils. A reference to one or two others will close this notice.

CASE.—Sarah Armstrong, married, aged 52, Feb. 15, 1843. Complains of much pain in the left deltoïd muscle, on attempting to put it into action. Is unable to raise the elbow in the least, nor can she carry the hand backwards.

The deltoïd is somewhat wasted. The other muscles of the arm appear unaffected, and she has perfect power over the fore-arm and hand.

She has been suffering from the complaint for the last year and a half. She attributes it to cold. She looks thin and rather out of health, but has no prominent general indisposition.

*Pil. hyd.* c̄ *colocynth.* gr. v. *o.* 3*tiâ nocte.*

*Infus. gent.* ℥x. *Trae ejusd.* ℨj.

*Magnes bicarb.* gr. x. *magnes. sulph.* ℥ss, *o.* 3*tio mane.*

*Emp. canths. humero.*

25th. No relief from the blister.

*Emp. belladonnæ humero. Adde misturæ vin. colchici.* ♏ x.

*P.* c̄ *pil.*

March 1st. *Rept. emp. lyttæ.*

11th. Mouth rather sore from pills—shoulder no better.

℞. *Pot. iodidi,* gr. v.

*Dec. sarzæ co.* ℥ij. *bis die.*

*Ext. col. co.* gr. x. *p. r. n.*

*Rept. emp. belladonnæ.*

April 18th. Suffers more pain in shoulder, and is worse altogether than she was—she is to have electricity applied to the shoulder twice a week.

23rd. Electrified to-day for the first time, as she did not like the idea of it before—much debility.

*Haust. quinæ bis in die.*

*Ext, col. co.* gr. x. *p. r. n.*

*May* 23. Has had electricity applied, but, at present, without benefit.

*June* 3. Can raise her arm a little, and but a little, from the side now. The moxa to be applied, so as to produce a superficial eschar.

24th. Much improved—has an ulcer on the front of shoulder, from the moxa, which discharges freely.

*July* 1. Can raise her hand to her head now, but cannot carry it backwards. *P.* c̄ *moxâ.*

22nd. Can carry her hand back a little way behind her head. *P.* c̄ *moxâ.*

The patient continued to attend up to the 9th September. Her general health was pretty good, but she had gone on taking the quina, as she was very weak. The power of raising the arm was, in a great degree regained; she could carry it much better, but not well, backwards and upwards. She complained much of general debility and looked out of health. Being so far recovered, she took a great dislike to the longer application of the moxa and discontinued her attendance.

The above case has been reported just as it was recorded in the out-patient book. Nearly at the time at which it was under my care, there was another female, of middle-age, with the same affection of the right shoulder. She derived great benefit from two applications of the moxa, when, alarmed at it, as very many people are, she too ceased to attend.

One of the most striking cases which has occurred to me was that of Colonel C. He had been exposed to weather in Ireland, and was of a family proverbial for gout. The left shoulder was first affected, and in spite of very active treatment, the use of that arm was temporarily lost. The other shoulder was then attacked, and the right arm, also, became paralysed. By long-continued counter-irritation, medicines adapted to the gouty diathesis, and a prolonged employment of vapour and shampooing baths, he gradually regained the power of the limbs, and when I saw him, the other day, had scarce a trace left of the complaint.

### II. Case of Cyst, containing Glairy Fluid on the Gum.

Ann Downey, aged 46, married, with three children, Nov. 4th, 1843. Cyst the size of the end of the thumb on the outer and upper surface of the lower jaw, occupying the places of the right canine and two bicuspid teeth. She has had it for six months, and it is increasing in size. The bicuspides have been drawn—the stump of canine tooth left—no teeth exist behind the cyst—general health good. This began to form shortly after having had a tooth extracted. Having punctured it with a grooved needle, and let out some yellowish glairy fluid, I passed a curved needle, armed with strong silk, through the cyst, and the ligature thread was loosely tied in, so as to act like a seton.

7th. Discharge of sanguineo-purulent matter from the interior of the cyst—no pain.

21st. Seton-thread removed, for stump of canine tooth to be extracted—which was done.

28th. Cyst still discharges, but much reduced in size, and the walls seem thickened—gum tender where tooth was drawn.

Dec. 8th. Seton-thread re-applied.

Feb. 14th. Continued seton in cyst till to-day, when it was withdrawn, as the cyst had quite disappeared, and its former situation was only marked by a line of thickening in the course of the thread. She subsequently presented herself well.

The case resembled ranula, in the character of the cyst, and the appearance of the fluid, save that it was yellower. It was treated with success, by what generally answers best for ranula—the seton.

### III. Rupture of the Inter-articular Fibro-Cartilage between the Lower Extremities of the Radius and Ulna.

This is not, so far as I am aware, a common accident. I have seen only two cases of it. The following presented itself at the hospital—the other occurred in private practice.

*Case* 1.—Ann Sargeant, aged 62, March 8, 1844. Five weeks ago she fell, with her whole weight, on her right hand. The hand "dropped" immediately after the accident, and was quite useless. Inflammation about the wrist supervened, but subsided after a week's surgical treatment. She is unable now to use her hand in the least degree, and there is considerable swelling remaining, as of effusion into the sheaths of the flexor tendons of the wrist. The shafts of both radius and ulna are perfectly entire, but there is unnatural mobility forwards and backwards of the lower ends of the ulna. The carpal extremities of the radius and ulna are separated from each other, the former projecting forwards, and the wrist presenting very much the appearance of oblique fracture of the lower end of the radius. To have Scott's bandage firmly applied to the wrist.

*Ext. col. co.* gr. v., *p. r. n.*

28th. Hand feels stronger, and she can use it a little. Scott's bandage still firmly applied.

April 20th. Much more power over her hand—still keeps the bandage on.

May 4th. Has removed the bandage, and uses her hand freely, but cannot lift anything very heavy with it. To pump cold water over it every day, and subsequently employ friction, with a stimulating liniment.

She did not attend any more, but she was accidentally seen a week or two ago. The wrist was still weak—the deformity inconsiderable.

*Case* 2.—A gentleman, when hunting, was thrown from his horse, and fell on the right hand and arm. He applied to the nearest surgeon, who examined the wrist, and pronounced that there was no fracture nor dislocation. On that evening, or the next day, he was seen by the family-surgeon, who considered that a fracture of the radius had occurred. He immediately came up to town to put himself under my care. When I saw him, the fore-arm and the wrist were so swollen, that it was inexpedient to meddle with the limb, and impossible to determine the nature of the accident. It had the *aspect* of oblique fracture of the lower end of the radius. By leeches and cold lotions the tumefaction was reduced in the course of a few days, and I was enabled to make an examination.

As in the former case, the lower end of the radius projected forwards, and this it was that occasioned the peculiar resemblance to oblique fracture of the lower end of the radius, where a projection always exists on the palmar aspect of the wrist. But the distinctive feature of the case was the prominence backwards of the lower end of the ulna, and its *unnatural mobility*. This proved, or seemed to prove, that the great bond of union between it and the radius, the inter-articular fibro-cartilage was torn. There was no fracture of bone.

With splints, bandages, and after three or four weeks' passive motion, with cold douche, and friction, all deformity was obviated, the ruptured cartilage appeared to unite, and perfect command of the wrist was restored.

I presume it must be admitted that, in these cases, the nature of the accident was such as I have supposed it to be. If so, the characteristic symptoms will be, great swelling—projection, towards the palmar aspect of the fore-arm, of the radius—unusual mobility of the lower end of the ulna.

### IV. Cachectic Ulceration of the Tongue.

The following is a not uncommon sort of case. It is occasionally mistaken for secondary venereal ulceration, and is probably confounded at times with malignant changes of structure.

*Case.*—Richard Green, aged 43, Dec. 6, 1843. The tongue is red, glazed, and irregularly fissured—the median line is occupied by a deep ravine of ulceration. The largest ulcer is situated on the right side of the tip of the tongue, not very deep, and of a yellowish brown colour—great induration surrounding the ulcers—that on the right side of the tip of the tongue is as large as a thumb-nail. There is not much pain—the articulation is thick, and the tongue feels too big for the mouth. General health tolerable, but has a cachectic aspect. The complaint began gradually three months ago. Has not had syphilis—has taken some mercury at different times.

*Ext. col. co.* gr. v. *o. n.*
*Haust. potassii iodidi bis die.*

9th. Speaks better, and ulcers look improved.
*Perstet.*

12th. Improving.

*Haust cinchonæ* ℥iss.

*Potass iodid.* gr. v. *bis die.*

*P.* c̄ *Pilâ.*

*Lotio zinci sulph.* gr. ij. *ad* ℥j. *pro linguâ.*

19th. Ulcer nearly healed—less induration. *P.*

29th. Great improvement.

*Jan.* 1, 1844. Ulcer at right side quite healed—considerable induration—tongue glazed, very moist, and clean.
*Perstet.*

12th. Ulcers healed—less induration—tongue irregular, but not so red and glazed.

22nd. Induration disappearing—no ulceration.

*Decoct. sarzæ co.* ℥ij. *bis quotidie.*

*Ext. col. co.* gr. v. *p. r. n.*

26th. General health very good—fat—tongue improves fast.

*Feb.* 2. Tongue is still rather hard, and of a glossy, smooth, red appearance. No ulcer in any part.—*P.*

*May* 11. Very slight induration of tongue now—it is touched with argent. nit. in the median fissure, where the ulceration was, as it feels tender.

*Perstet.*

*April* 10. Has continued the sarsaparilla up to the present date. There is still a little hardness left in the tongue, but it is very slight, and he is to be discharged.

The progress towards cure in the preceding case was slow, as it generally is. But in some instances (they are rare exceptions) the patient recovers with rapidity.

A gentleman consulted me some time ago with the same affection of the tongue as the preceding patient. It had existed for three or four years, and he had tried many remedies in the country. He was dyspeptic, and had taken some mercury, but had nothing which could be fairly construed into secondary syphilitic symptoms. I prescribed sarsaparilla with the iodide of potassium—mild aperients, mercury being excluded from their composition—a very regulated diet and regimen—and the occasional application of the nitrate of silver to the fissures, which were daily washed with a lotion of bark and myrrh. In the course of two or three weeks the tongue was well. He had a relapse many months afterwards, but got well again with the same remedies.

The distinctive characteristics of cachectic ulceration of the tongue, are the irregular fissures—the yellowish ulceration, generally deepish, sometimes superficial—the induration limited to the vicinity of the ulcers—the glazed, red, and raw looking surface (not, however, constant) of the organ—the absence of any indication of malignant disease—the indifferent tone of the general health—the persistence of the complaint without other symptoms, save, perhaps, those which are common attendants on cachexia.

This condition of tongue seems to me to occur in two states of system, differing in some respects, though allied in others. On the one hand, it is found in those who have been reduced to cachexia by mercury. It may then be combined, preceded, or followed, by the other symptoms of mercurial cachexia—rupia, ecthyma, nodes, phagedænic and serpiginous ulcerations on the skin and in the throat.

On the other hand, this condition of tongue obtains in those whose systems are debilitated by dyspepsia, especially by that form of it attended with morbid sensibility and irritability of the gastro-intestinal mucous membrane. We shall be the less disposed to wonder at this, when we remember how readily, in all persons, slight derangements of digestion, lead to small, yellow, painful, and perhaps, obstinate ulcers of the tongue, and cheeks, and lips. The continuity of tissue, and of function readily enough explains the liability.

The affection, however it may have been induced, always seems to require tonics in some shape or other, regimen, diet, and all that can invigorate the constitution. Sarsaparilla has generally proved highly useful—the iodide of potassium not seldom so—the ammonio-citrate of iron I have beneficially combined with it—in short, the alterative tonics are requisite. Of course, the secretions must be regulated, and in those cases dependent on derangement of the digestive organs, small doses of mercury may be serviceable. But I should doubt the propriety of its administration, nor would I readily be induced to exhibit it, in such as may be found to display a connexion with the form of cachexia so often due to, and so frequently aggravated by that medicine.

---

### NEW YORK HOSPITAL.

### Renal Disease. By J. A. Swett, M.D.

In our New York contemporary for July last, we have a report from Dr. Swett, of eight cases of renal disease, which we shall briefly notice in this article.

*Case* 1.—Mary Burke, 31 years of age entered the Hospital, 27th Nov. 1843. In the latter end of March preceding, after parturition, began to complain of dull pain in the loins and limbs—with pale urine—costive bowels—and, soon afterwards œdematous feet, gradually extending to the abdomen. This state, with little variation, continued till her entry into hospital. The urine now evinced much albumen—tongue coated in centre, red and glossy at the sides—appetite good—bowels relaxed—skin dry—pulse 100 and weak. Morphia at night, with decoct. querci. Dec. 5. Passes from five to six pints in the 24 hours—bowels dysenteric—cough. Expectorants—morphia—nitrate of silver with opium. Dec. 24. Seized with severe pain in the lumbar region—epigastric tenderness—vomiting of dark matter—diarrhœa. Five grains of blue pill thrice a day. Jan. 1, 1844, seized with convulsions—death in a few hours.

*Post-mortem.*—Pneumonia of the right lung—liver presented the first stage of cirrhosis—kidneys enlarged, of a uniform pale yellow colour, granulated, soft, and flabby—cortical portion hypertrophied—tubular portion livid red colour—pelves natural.

The second Case was one of Phthisis in a young man, where no renal disease was suspected, and who died suddenly. Disease of the kidney as well as of the lungs was found.

*Case* 3.—This was also a case of phthisis, in a man, 37 years of age, where kidney disease supervened, exhibiting œdema, ascites, dyspnœa, coagulable urine. He had no pain in the loins. Antiphlogistic treatment was adopted. Blood drawn was highly buffed. Cupped on the loins—warm-bath—low diet. In three weeks the dropsy had increased—cough very troublesome—cupped again on the loins, and the bath and antiphlogistic means continued. This treatment lessened his sufferings, diminished the albumen; but increased the dropsy. Diuretics were tried and abandoned; and diarrhœa and erysipelas terminated life.

*Post-mortem.*—Heart and pericardium healthy—lungs tuberculated in various stages—ulceration of the larynx—liver pale and granulated—kidneys rather enlarged, pale and flabby, but mottled with red spots—no granulations were found.

*Case* 4.—Elisha Lynch, aged 24 years, was admitted on the 27th January. Had had two attacks of intermittent fever. About Christmas previously noticed swelling of the ancles, which increased and reached the abdomen, with palpitation of the heart, occasional vomiting, and cough.

On admission, the anasarca was general—abdomen much distended—countenance pale—heart's action natural—mucous râle posteriorly in the lungs—urine abundantly albuminous—micturition—had had pain in the loins formerly—tenderness now over the right kidney. Supertartrate of potash with digitalis, 6tis horis. Purged and nauseated by the medicine without relief to the dropsical symptoms. Decoct. apocyni canab. a wineglassful 6tis horis.* This was con-

---

* Indian hemp, which grows plentifully in America, and is much used as evacuant by mouth and bowels, especially in dropsy.

tinued for five weeks, the effect of which was four or five motions daily, without pain or griping, but without any diminution of the dropsical symptoms, or increase of urine. Bronchitis troublesome, with pain and dizziness in the head. Cupping, warm-bath, low diet. Complains of pain in the region of the left kidney. Mercury was now given till the gums were affected; but no relief followed. Then the oxymuriate of mercury with bark was given, which acted decidedly as a diuretic, and removed the dropsical effusions. In three weeks after this plan was adopted he was discharged cured.

We doubt whether this case could properly come under the term *diseased* kidney? Does organic change of structure in that organ ever admit of cure? May there not be *disordered function* of the kidney, with albuminous urine, and dropsy, and yet no diseased structure of the kidney or any other organ? We have little doubt that this last condition was that of Elisha Lynch.

*Case* 5.—*Bright's Disease treated by Diuretics.*—Nathan Beckwell, a man of colour, aged 28 years, came into Hospital 30th Jan. 1844. Had contracted rheumatism twelve months previously, which continued two months. During the past summer had head-aches, night-sweats, and emaciation—more recently had cough, dyspnœa, palpitation, and diarrhœa. Has now anasarca, irritability of bladder, and some effusion into the abdomen. Pulse 90, skin dry—urine slightly acid and five pints daily—deposits albumen copiously when tested—no pain in the region of the kidneys. Two drachms of supertart. potass. and 15 drops of tinct. digitalis, thrice a day.

*March* 9.—Urine materially increased—sometimes to 12 pints in the 24 hours, attended with diminution of the dropsy—cessation of cough—no tenderness over the kidneys. The urine, however, is still albuminous, and began now to decrease in quantity. The diuretics were now discontinued, and a quarter of a grain of elaterium every four hours.

*March* 21.—The elaterium purged, griped, and vomited, it was therefore omitted. The decoct. apocyni also disagreed. The urine was now four pints in the 24 hours, but still albuminous. The legs were œdematous; but his general health was improved: and he requested to leave the hospital on the 19th April.

Unless albuminous urine and dropsy be unequivocal proofs of diseased kidneys, this case leaves us in doubt.

*Case* 6.—This patient was a Swede, aged 33 years, of good constitution, who entered the Hospital, 15th Nov. 1843. He had had chronic rheumatism in the hospital at Hamburg, for six weeks, and was salivated there—he has now rheumatic pains in the shoulders and back—œdema of the lower extremities—tongue coated—pulse natural—bowels regular—tenderness of hypochondria—dyspnœa. Took hydriodate of potash, which relieved the pains a little—he then had purgatives.

*Jan.* 17.—Had been examined several times. The urine always coagulable—rather acid—and about two quarts in the day. Pain in the loins—pulse natural—skin moist—digestion good. He had been still using the pulv. jalap. comp. which now failed. Elaterium twice a day. This did not diminish the œdema, and was omitted on the 27th Jan. when the pulvis purgans, with tinct. digitalis were substituted. Feb. 11. The urine very albuminous, and the other symptoms no better. Ordered an eighth of a grain of oxymuriate of mercury, with ʒj. tinct. cinchonæ, ter die. This treatment was continued till the 11th of May, when he was last seen. His strength was decreasing—the urine highly albuminous, and below the natural specific gravity—some œdema of the ankles. In fact, he was in anything but a satisfactory condition, and the probability is that he has since died.

Two other cases of "Bright's disease" are recorded, but we can do little more thak give a resumé of them.

*Case* 7.—A man of colour, aged 37, entered the Hospital 27th Dec. 1843. After severe rheumatism the legs became œdematous—pain in the loins—eight pints of urine daily, urine albuminous. He was treated by purgatives and elaterium, which diminished the œdema and the quantity of urine; but he thought himself weaker, and the albuminaria continued. 11th May. Little alteration, and he was now lost sight of.

The eighth case was one where there was hypertrophy of the heart, with dropsical effusions, and albuminous urine. He was treated with diuretics and purgatives, including elaterium. The case is not concluded.

In these cases the dropsical effusions were the most constant attendants on albuminaria. There were few evidences of inflammatory action going on, although the pulse was sometimes accelerated. The Reporter dwells much upon a "smoky appearance" in the urine as indicative of albumen. This phenomenon prevents the bottom of the pot from being clearly seen. The specific gravity was uniformly less than in health. The urine was alkaline in only one case. The causes of the disease could not be ascertained. Rheumatism not unfrequently preceded the attack; and only one patient confessed himself to be intemperate. In respect to treatment, Dr. S. candidly confesses that success was very indifferent. Dropsical swellings were occasionally reduced, but the albuminous state of the urine continued in all.

---

### Dangers Incidental to Operations about the Rectum. By Dr. Watson, Surgeon to the New York Hospital.

Our New York contemporary for July last, contains a useful paper on the above subject by a practical hospital surgeon. In the course of the last few years Dr. W. has witnessed four fatal terminations of operations about the anus—one after excision of mucous membrane—and three after ligatures—none of them with hæmorrhage. We shall glance at some of these.

*Case* 1.—A seaman aged 32 years, admitted into the hospital 10th May, 1841, for prolapsus ani and some stricture of urethra. The general health was not good. A prolapsus took place after each evacuation, but was easily reduced in general, except when inflamed. The stricture soon gave way to the armed bougie, and afterwards the prolapsus was included in ligatures and cut away. The patient suffered a good deal and had strangury; but appeared to be doing well for a couple of days. On the fourth day, however, after a relaxed motion, great prostration of strength occurred, and he died the same evening. Secondary abscesses or purulent deposits were found in the lungs.

*Case* 2.—"On the 6th June, 1843, I was requested to see Mr. B. a merchant from Kentucky, about 30 years of age, who was suffering from extensive disease of the right elbow and fore arm, and a similar affection of his right ankle, which had occurred suddenly a few days previous, and appeared to be the indirect result of an operation which he had recently undergone for the removal of hemorrhoidal tumors.

"Just before the operation he had arrived here on business, and, with the exception of the hemorrhoids, in the enjoyment of tolerable health. For some time previous, however, he had suffered from a slight stricture in the urethra, which had affected his general system, and induced a deranged condition of his digestive organs; but of these symptoms he was not then complaining.

"On the 20th of May, the Gentleman with whom I saw this patient in consultation, had applied a silk thread around the base of a small hemorrhois, which, in a few days, sloughed, and left the patient comfortable. On the 29th, a second hemorrhois having appeared, and the patient having completely recovered from the effects of the first operation, a ligature was applied around the base of this little tumor also. On the 2d of June, the patient had a chill, followed by severe pain in the right ankle, and soon after, by redness and tumefaction about this part. In the course of the next twenty-four hours, the right elbow and arm as far as the shoulder, were similarly attacked. Notwithstanding the most prompt and judicious treatment, the application of nitrate of silver, blistering, and free scarification, and the internal administration of tonics, stimulants, and anodynes, for allaying pain and supporting the patient's strength, these local affections rapidly progressed to suppuration.

"When I first saw the patient, the whole arm to the shoulder was enormously swollen, boggy, and injected with serous and purulent deposits. It had been deeply and freely scarified; and, from a puncture just below the elbow, pus and sloughy cellular tissue were issuing. The whole had been enveloped in a poultice. The ankle and foot were in the same condition, except that the integuments had not yet given way to the accumulated fluids beneath. The patient was apparently free from arterial excitement; his pulse 88, and of moderate volume; his skin of natural temperature; his tongue clean, moist, and of natural color; his intellectual faculties somewhat subdued and heavy, but without delirium. He had no internal pain, with the exception of a dull, uneasy sensation under the short ribs, on the right side; and this he attributed to having lain on the part. He had no special uneasiness about the anus, no tympanitis, no difficulty in evacuating the bladder, although he had suffered slightly from strangury, and the accumulation of gas in the bowels, for a short time after the application of the ligature. He was on the use of nourishing diet, with porter and repeated doses of Dover's powder. To these I added a grain of quinine every four hours, and small doses of camphor emulsion every hour, and suggested the use of brandy in case the patient should have any symptoms of sudden collapse before the next visit.

"*June 8th at* 8 *o'Clock* A.M.—The patient's face is bathed in perspiration; his features are sunken; his left hand is cold, clammy, and purplish; his pulse 130 and extremely feeble; his tongue dry, cracked on the surface, and red at the edges; his intellect is clear; he is free from pain; but he has a constant hiccough which has continued since last evening. He had been on the use of small and repeated doses of brandy all night. His condition was too critical to admit of any examination of the arm or ankle. His stimulants were continued: but he died about mid-day. No post-mortem examination could be obtained."

*Case* 3.—In May, 1842, Dr. Mott operated on a gentleman of middle-age and good health, for prolapsus ani. He suffered but little from the ligature; but on the second day he presented symptoms of collapse, and towards the close of the fourth day he sunk, with all the symptoms of typhus fever. No examination could be obtained.

The writer of this notice nearly lost his life by the excision of piles, which was followed by abscesses requiring twice the operation for fistula. Still we must not be deterred by some fatal results; but we should always bear in mind that they may and do occasionally happen. Every precaution, therefore, should be taken to get the general health of the patient into as good a state as possible before the operation.

